# Feasibility study of mobile phone photography as a possible outcome measure of systemic sclerosis-related digital lesions

**DOI:** 10.1093/rap/rkac105

**Published:** 2022-12-07

**Authors:** Adrian K Davison, Graham Dinsdale, Paul New, Joanne Manning, Helen Patrick, Vicky P Taxiarchi, William G Dixon, Andy Vail, Andrea K Murray, Mark Dickinson, Christopher Taylor, Ariane L Herrick

**Affiliations:** Division of Musculoskeletal and Dermatological Sciences, The University of Manchester, Manchester, UK; Division of Musculoskeletal and Dermatological Sciences, The University of Manchester, Manchester, UK; Northern Care Alliance NHS Foundation Trust, Manchester Academic Health Science Centre, Manchester, UK; Division of Musculoskeletal and Dermatological Sciences, The University of Manchester, Manchester, UK; Division of Musculoskeletal and Dermatological Sciences, The University of Manchester, Manchester, UK; Northern Care Alliance NHS Foundation Trust, Manchester Academic Health Science Centre, Manchester, UK; Division of Musculoskeletal and Dermatological Sciences, The University of Manchester, Manchester, UK; Centre for Biostatistics, Manchester Academic Health Science Centre, The University of Manchester, Manchester, UK; Division of Musculoskeletal and Dermatological Sciences, The University of Manchester, Manchester, UK; Northern Care Alliance NHS Foundation Trust, Manchester Academic Health Science Centre, Manchester, UK; Centre for Biostatistics, Manchester Academic Health Science Centre, The University of Manchester, Manchester, UK; Division of Musculoskeletal and Dermatological Sciences, The University of Manchester, Manchester, UK; Northern Care Alliance NHS Foundation Trust, Manchester Academic Health Science Centre, Manchester, UK; Photon Science Institute, Manchester Academic Health Science Centre, The University of Manchester, Manchester, UK; Photon Science Institute, Manchester Academic Health Science Centre, The University of Manchester, Manchester, UK; Division of Informatics, Imaging & Data Sciences, Manchester Academic Health Science Centre, The University of Manchester, Manchester, UK; Division of Musculoskeletal and Dermatological Sciences, The University of Manchester, Manchester, UK; Northern Care Alliance NHS Foundation Trust, Manchester Academic Health Science Centre, Manchester, UK; NIHR Manchester Biomedical Research Centre, Central Manchester NHS Foundation Trust, Manchester Academic Health Science Centre, Manchester, UK

**Keywords:** digital ulcers, SSc, smartphone photography, imaging

## Abstract

**Objective:**

Clinical trials assessing systemic sclerosis (SSc)-related digital ulcers have been hampered by a lack of reliable outcome measures of healing. Our objective was to assess the feasibility of patients collecting high-quality mobile phone images of their digital lesions as a first step in developing a smartphone-based outcome measure.

**Methods:**

Patients with SSc-related digital (finger) lesions photographed one or more lesions each day for 30 days using their smartphone and uploaded the images to a secure Dropbox folder. Image quality was assessed using six criteria: blurriness, shadow, uniformity of lighting, dot location, dot angle and central positioning of the lesion. Patients completed a feedback questionnaire.

**Results:**

Twelve patients returned 332 photographs of 18 lesions. Each patient sent a median of 29.5 photographs [interquartile range (IQR) 15–33.5], with a median of 15 photographs per lesion (IQR 6–32). Twenty-two photographs were duplicates. Of the remaining 310 images, 256 (77%) were sufficiently in focus; 268 (81%) had some shadow; lighting was even in 56 (17%); dot location was acceptable in 233 (70%); dot angle was ideal in 107 (32%); and the lesion was centred in 255 (77%). Patient feedback suggested that 6 of 10 would be willing to record images daily in future studies, and 9 of 10 at least one to three times per week.

**Conclusion:**

Taking smartphone photographs of digital lesions was feasible for most patients, with most lesions in focus and central in the image. These promising results will inform the next research phase (to develop a smartphone monitoring application incorporating photographs and symptom tracking).

## Graphical Abstract

**Graphical Abstract:**
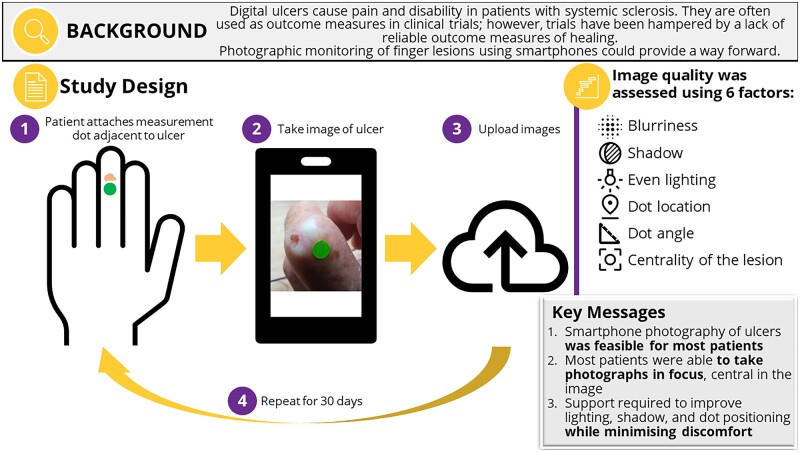

Key messages

- Smartphone photography of finger lesions was feasible for most patients with SSc.
- The majority of patients were able to take photographs in focus, with the lesion central in the image.
- Smartphone photography has potential as an outcome measure in clinical trials of digital ulceration.

## Introduction

Much of the morbidity of the multisystem CTD SSc (also termed scleroderma) relates to painful, disabling digital ulcers (ulcers of the fingers and sometimes toes), which develop in ∼50% of patients [1–3]. Digital ulcers can be very difficult to heal, and they result in major disability (including work disability [4, 5]), with loss of hand function and a major negative impact on quality of life [6, 7].

Although some treatments have beneficial effects [8, 9], better, more effective treatments are required. Disappointingly, several multicentre, multinational studies reported in the last 6 years have failed to reach their primary endpoint [10–12], although some showed trends in favour of active treatment. It was suggested that one reason why the SEDUCE study, comparing sildenafil with placebo [11], failed to meet its primary endpoint (time to ulcer healing) was because of ‘inaccurate evaluation of time to healing’, given that patients were assessed only at intervals of 4 weeks. Subsequent to the trial of oral treprostinil [12], a subset of patients (studied retrospectively) experienced an increase in digital ulcer burden after discontinuation of treprostinil [13], implying that treprostinil had conferred benefit. It is very possible that the failure to show benefit in randomized controlled trials of SSc-related digital ulceration has been attributable to the inadequacy of the primary outcome measure. This is because the primary outcome measure has typically involved clinician classification of lesions as ulcers, which we and others have shown to be unreliable [14–16], and because the outcome has been measured at sparse intervals.

Until now, lack of reliability of digital ulcer definition has made it very difficult (if not impossible) to track ulcer/lesion trajectories. To avoid confusion, we shall henceforth use the term (digital) lesion. Photographic monitoring of digital lesions using smartphones could overcome this difficulty by allowing objective (and frequent) analysis of lesions. Nowadays, 9 in 10 people in the UK carry a smartphone [17], providing the ideal platform to capture photographs/images of finger lesions repeatedly over time. Our objective was to assess the feasibility of patients collecting high-quality mobile phone images of their digital lesions, as a first step in a programme of research to develop a smartphone-based outcome measure for use in clinical trials. This built upon our experience from a small pilot study [18]. Feasibility was assessed using a combination of the number and quality of images collected, and patient feedback.

## Methods

### Patients

Patients fulfilling the 2013 criteria for SSc [19] and with one or more finger lesions were recruited. All were attending a single tertiary centre for SSc and were >18 years of age. The study was approved by London-Chelsea Research Ethics Committee, and all patients signed informed consent. All patients were asked to complete a smartphone questionnaire (see Supplementary Data S1, available at *Rheumatology Advances in Practice* online) asking about their smartphone use/experience.

### Development of imaging protocol

This was developed with input from a patient user group attended by five patients, all of whom had SSc and had experienced one or more finger lesions. Topics discussed included how frequently to image lesions and how best to do this given that many patients with SSc have impairment of hand function. Suggestions included the following: using the rear-facing (standard) camera to photograph one hand while operating the phone with the other; using the front-facing camera with the phone lying flat on a surface facing upwards and with the hand to be photographed above it; resting the hand to be imaged on a flat surface to reduce motion artefact (when using the camera in standard mode); and asking a friend or partner to take the images. The imaging protocol can be viewed in Supplementary Data S2, available at *Rheumatology Advances in Practice* online. In brief, patients were instructed to photograph one or more finger lesions each day for 30 days using their smartphone, at the same time each day and ideally in the same location. Instructions on how to take the photographs were given either face to face or [especially relevant during the coronavirus disease 2019 (COVID-19) pandemic] remotely. Patients could take photographs using either the rear-facing (standard) camera on the phone or using the front-facing camera. An adhesive dot, placed adjacent to the lesion(s), provided a 6 mm reference scale for extracting accurate measurements. Images were uploaded by each patient to a secure Dropbox folder.

### Assessment of images

A visual inspection of all images was undertaken by the same observer (A.K.D.) using six subjective criteria: (i) blurriness, by checking for the focus of the photographed image, specifically around the lesion of interest; (ii) shadow, by assessing whether any shadowing was present over the lesion, for example from the patient’s phone over the lesion; (iii) evenness of lighting, by looking at light levels in the image and concluding that there was uneven lighting if there was a large fall-off in light or colour; (iv and v) the measurement dot location and angle, which were closely related, with the dot location being good if the dot was placed adjacent to the lesion and on the same plane, whereas the dot angle assessed how well patients could photograph the dot while maintaining the circular shape; and (vi) positioning of the lesion in the centre of the image.

### Patient feedback

After image collection, patients were asked to complete a feedback questionnaire covering 14 items (see Supplementary Data S3, available at *Rheumatology Advances in Practice* online). The first seven questions asked about ‘following the imaging instructions’, with questions 1–6 being answered with a numerical rating scale from 1 (very easy) to 10 (very difficult). There was also an open-ended response box for patients to provide additional comments on the imaging instructions. Questions 8–12 asked about the physical experience of using their smartphone to take photographs. Question 8 was a single response option question on how the phone was handled. Questions 9–11 were on a numerical rating scale from 1 (very easy) to 10 (very difficult). Question 12 was an open-ended response box for any other comments on the physical or practical aspects of taking the photographs. The final two questions, 13 and 14, were general questions on the photograph submission frequency and willingness of participants to record finger lesions using photographs in future studies.

## Results

### Patients

Twelve patients (10 female, median age 55 years, range 37–72 years) were recruited between February 2021 and July 2021. Seven patients were recruited remotely and five at the outpatient clinic. The median duration of RP was 11.4 years (range 2–30 years), and the median disease duration (from the onset of the first non-RP clinical manifestation) was 10.6 years (range 1–27 years). Of the 12 patients, 10 completed the smartphone usage questionnaire at the beginning of the study. All 10 indicated that they used their phone for calls, texting and taking pictures, with 9 also using it for browsing the Internet and using other applications. Nine patients reported that their ulcers impaired their ability to use their smartphone ‘not at all’, or only ‘a little’, with one patient stating that her ability was impaired ‘a lot’.

### Image collection

The 12 patients returned a total of 332 photographs from 18 lesions: eight patients photographed one lesion, two patients two lesions, and two patients three lesions. Eleven lesions were located on the fingertip, three on the extensor surface, two on the nailbed, and two in other locations. Six of the lesions were located on the left hand, 11 on the right, and one unknown. The median number of photographs returned by each patient was 29.5 [interquartile range (IQR) 15–33.5], with a median of 15 photographs per lesion (IQR 6–32). The total possible number of images was 540 if all patients took a photograph each day for 30 days. Twenty-two photographs were classed as duplicates (taken on the same day). There were therefore 230 missing photographs, giving a final submitted image proportion of 57.4%. For duplicate images, the first usable image was used.

### Image quality

**Figure 1.**
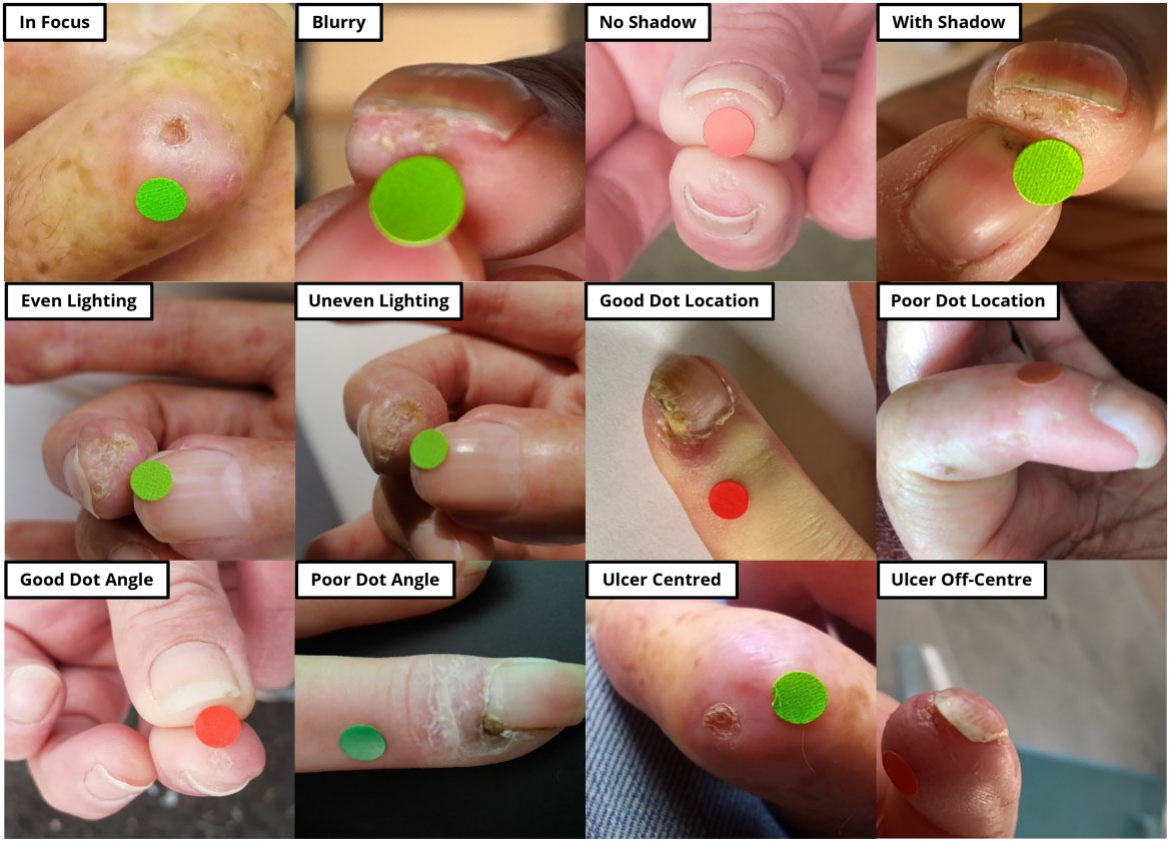
Examples of images to show good and less good photographs in terms of the six criteria of blurriness, shadow, lighting, dot location, dot angle and position

Fig. 1 shows examples of lesions as assessed according to the six criteria of blurriness, shadow, lighting, dot location, dot angle and position. Two hundred and fifty-six (77%) of the photographs were sufficiently in focus; 268 (81%) had some shadow; lighting was even in 56 (17%); dot location was acceptable in 233 (70%); dot angle was ideal in 107 (32%); and the lesion was centred in 255 (77%).

### Patient feedback

Of the 12 patients recruited, 10 completed the post-study feedback questionnaire. The main results are shown in Table 1, indicating that, overall, the patients rated taking photographs as easy. Six of 10 responders stated that they would be happy to take images every day, three would prefer to submit photographs two or three times per week, and one stated that they believed that photographing their lesions once per day was ‘far too often’ and would prefer once a week or less. All respondents held their phone themselves when taking photographs.

**Table 1.** Results from the feedback questionnaire, which was completed by 10 patients

**Post-study questions** **[Likert scale from 1 (very easy) to 10 (very difficult)]**	Median (interquartile range)
Remembering to take photographs of digital ulcers?	4 (3–6)
Taking photographs at the same time every day?	4 (3–6)
Taking photographs in the same place every day?	3 (2–4)
Keeping the environment and lighting the same each time?	4.5 (2–5)
Making sure hands were in the same condition each day?	2 (2–2)
Overall experience of using the mobile phone to photograph your digital ulcer?	3 (3–4)
Holding the phone while imaging?	4 (3–7)
Pressing the button or screen to take an image?	5.5 (3–7)
Getting a good clear image of your digital ulcer?	5 (4–5)

The reason for these appearing not to be phrased as proper questions is due to them being taken directly from the end of feedback questionnaire (Supplementary Data S3). Here, there is leading text that forms the whole question. It states: “In each case, circle the number that best describes your experience”, which can lead into each of the 9 questions in this table.

## Discussion

We have shown that taking smartphone photographs of digital lesions is feasible for the majority of patients, although missing images and patient feedback suggested that it is challenging for patients to sustain daily collection for a month. Most patients were able to take photographs in focus and with lesions central in the image. The fact that almost all patients intimated that they would be willing to record images at least one to three times per week is very encouraging, because in a clinical trial setting it is likely that once- or twice-weekly photographs would be sufficient to allow an accurate estimate of ulcer healing and would certainly be much more accurate than has hitherto been possible, when intervals between visits tend to be 4 weeks or less frequent.

The study has highlighted areas where patients will require support to maximize photographic image quality, specifically the importance of even lighting, avoiding shadow and trying to ensure that photographs are taken at a satisfactory angle. Taking photographs can be challenging for patients with SSc, many of whom have impaired hand function. After this feasibility study was completed, we convened a further focus group and discussed with patients the limitations of taking some of the photographs. Clear guidance for future studies will include recommendations for lighting conditions. Positioning of the mobile phone camera is always likely to be difficult for some patients. Use of a tripod might help, although this might also prove problematic when hand function is impaired.

Our study had limitations. The number of patients recruited was lower than intended because of the COVID-19 pandemic, with fewer patients attending hospital than in previous years, although this problem was overcome for some patients by remote recruitment and training. Also, because of the difficulties in recruiting patients, we included some finger lesions (e.g. those bordering on severe pitting as opposed to active ulcers) that would not generally be perceived as ulcers [16] and that would therefore not qualify for inclusion in a clinical trial of digital ulceration. We felt that this approach was justified in the context of a feasibility study, the main purpose of which was to assess whether patients with SSc and severe digital vasculopathy could acquire and upload photographic images of their fingers. Although patient numbers were small, their clinical characteristics suggest that they were comparable to larger cohorts of patients with SSc-related digital lesions [1, 2]. It was outside the remit of this feasibility study to investigate associations between photographic appearances and patients’ symptoms (including symptoms of infection) or to examine change in photographic appearances (and lesion size) over time; these are currently being assessed in an ongoing programme of work.

The encouraging results of this feasibility study will inform the next phase of this research, which is to develop a smartphone application (app) for monitoring finger lesions and which will serve as an outcome measure to facilitate clinical trials of SSc-related digital ulceration. The next steps are to develop methods of extracting data from the images to track healing status reliably and automatically, and to combine (in the app) photographic images with patient-reported outcome measures. Such an app could be used for clinical practice and for research by integrating mobile phone images into clinical care, as we have done successfully for symptom tracking in RA [20]. This might allow clinicians to advise on management with a clearer picture of how lesions have changed through time or even without the need for a face-to-face consultant, which is especially relevant for patients living long distances from the hospital and during the COVID-19 pandemic.

## Supplementary Material

rkac105_Supplementary_Data

## Data Availability

The data underlying this article cannot be shared publicly for the privacy of individuals who participated in the study and owing to being outside of the remit of the ethics application of the study.
