# Supplementary material for: Feasibility study of mobile phone photography as a possible outcome measure of systemic sclerosis-related digital lesions
Source: Rheumatol Adv Pract. 2022 Dec 7;6(3):rkac105. doi: 10.1093/rap/rkac105 (PMC9757677; doi:10.1093/rap/rkac105)
Supplement: rkac105_Supplementary_Data [file rkac105_supplementary_data.zip › S2_Imaging_protocol.docx]

**Development of a measuring app for finger lesions as an outcome measure for systemic sclerosis-related digital ulceration (SALVE: Scleroderma App for Lesion VErification) (Study 1)**

**Digital ulcer imaging protocol v1.0**

**Objective:** a simple set of instructions for patients to follow to allow them to capture high-quality photographs of their digital (finger) ulcers using their smartphone camera and app.

**Scope:** for use by study participants testing a smartphone app as part of the SALVE (Scleroderma App for Lesion VErfication) project.

**Assumptions:**

1. Participant has a smartphone with built-in camera, and has had the SALVE study app installed onto the device.
2. Participant has one or more digital ulcers.

**Imaging timing and frequency:** participants will be asked to image each digital ulcer once per day for the duration of the study. It will be explained to them that this should ideally occur at the same time each day (e.g. just before bed time). An app notification will alert the user when the next image is due.

**Environment:** the photographs should, where possible, be taken in the same location every day. This is likely to be in the participants own home, and should be consistently well lit and as free from visual clutter as is possible. It is recommended that there is a flat surface, such as a table or counter top on which the participant can rest their hands while imaging – this is to reduce the possibility of motion artefacts (blur).

**Considerations before imaging:** (to be discussed with the participant to take account of daily routines etc.) To help with consistency in imaging, it is best if the hands are in the same “state” at each imaging session. This should include time since last washed, application of hand creams or treatments, and removal or application of dressings on digital ulcers. If dressings are not to be removed on a particular day, then the participant can select “imaging not possible today” in the app, to skip the session.

**Taking the photographs:** Users have two options for photographing their digital ulcers: (1) is to use the rear (standard) camera on the phone, and photograph one hand while holding/operating the phone with the other; (2) is to use the front-facing camera (screen side/selfie camera) with the phone lying flat on a surface facing upwards. This second option is perhaps best for those participants with issues with hand function. This choice will be set at the start of the study, but can be changed in the app settings menu.

On opening the app, the user presses the “take photograph” button and is immediately taken to the live camera view. The user should position the camera and their digital ulcer in such a way that the ulcer is in clear focus and can be held in a steady position. The user can then press the shutter button to take the image. Once the image is captured it can be accepted (“Image OK”) or rejected (“try again”) by the user.

The participant repeats the process for each digital ulcer they wish to include in the study.
