# Supplementary material for: Feasibility study of mobile phone photography as a possible outcome measure of systemic sclerosis-related digital lesions
Source: Rheumatol Adv Pract. 2022 Dec 7;6(3):rkac105. doi: 10.1093/rap/rkac105 (PMC9757677; doi:10.1093/rap/rkac105)
Supplement: rkac105_Supplementary_Data [file rkac105_supplementary_data.zip › S3_End_of_study_feedback_questionnaire.docx]

**Development of a measuring app for finger lesions as an outcome measure for systemic sclerosis-related digital ulceration (SALVE: Scleroderma App for Lesion VErification) (Study 1)**

**End of Study 1 Feedback Questionnaire**

The following questionnaire asks about your experiences of imaging your digital lesions/ulcers using your mobile phone**.**

Patient ID:

**Following the imaging instructions**

For points 1 to 7, please use the scales below to describe your experience of taking pictures of your digital lesions/ulcers. In each case, circle the number that best describes your experience.

1. Remembering to take photographs of digital lesions/ulcers?

| **1** | **2** | **3** | **4** | **5** | **6** | **7** | **8** | **9** | **10** |
| --- | --- | --- | --- | --- | --- | --- | --- | --- | --- |
| **Very easy** |  |  |  |  |  |  |  |  | **Very difficult** |

1. Taking photographs at the same time every day?

| **1** | **2** | **3** | **4** | **5** | **6** | **7** | **8** | **9** | **10** |
| --- | --- | --- | --- | --- | --- | --- | --- | --- | --- |
| **Very easy** |  |  |  |  |  |  |  |  | **Very difficult** |

1. Taking photographs in the same place every day?

| **1** | **2** | **3** | **4** | **5** | **6** | **7** | **8** | **9** | **10** |
| --- | --- | --- | --- | --- | --- | --- | --- | --- | --- |
| **Very easy** |  |  |  |  |  |  |  |  | **Very difficult** |

1. Keeping the environment and lighting the same each time?

| **1** | **2** | **3** | **4** | **5** | **6** | **7** | **8** | **9** | **10** |
| --- | --- | --- | --- | --- | --- | --- | --- | --- | --- |
| **Very easy** |  |  |  |  |  |  |  |  | **Very difficult** |

1. Making sure hands were in the same condition (e.g. no recent hand cream or hand washing) each day?

| **1** | **2** | **3** | **4** | **5** | **6** | **7** | **8** | **9** | **10** |
| --- | --- | --- | --- | --- | --- | --- | --- | --- | --- |
| **Very easy** |  |  |  |  |  |  |  |  | **Very difficult** |

1. Overall experience of using the mobile phone to photograph your digital lesion/ulcer?

| **1** | **2** | **3** | **4** | **5** | **6** | **7** | **8** | **9** | **10** |
| --- | --- | --- | --- | --- | --- | --- | --- | --- | --- |
| **Very easy** |  |  |  |  |  |  |  |  | **Very difficult** |

1. Do you have any other comments about the imaging instructions used in this study? Please do not include any identifiable data such as your name when completing the comments sections.

**Taking the photographs**

For points 8 to 11, please use the scales below to describe your experience of physically taking the photographs with your mobile phone. In each case, circle the item or number that best describes your experience.

1. Did you mainly hold the phone while taking photographs, did you place it on a surface and use the front-facing camera, or did someone lese take the photographs for you?

| Held the phone | Placed on surface | Someone else helped me |
| --- | --- | --- |

1. Holding the phone while imaging?

| **1** | **2** | **3** | **4** | **5** | **6** | **7** | **8** | **9** | **10** |
| --- | --- | --- | --- | --- | --- | --- | --- | --- | --- |
| **Very easy** |  |  |  |  |  |  |  |  | **Very difficult** |

1. Pressing the button or screen to take an image?

| **1** | **2** | **3** | **4** | **5** | **6** | **7** | **8** | **9** | **10** |
| --- | --- | --- | --- | --- | --- | --- | --- | --- | --- |
| **Very easy** |  |  |  |  |  |  |  |  | **Very difficult** |

1. Getting a good clear image of your digital lesion/ulcer?

| **1** | **2** | **3** | **4** | **5** | **6** | **7** | **8** | **9** | **10** |
| --- | --- | --- | --- | --- | --- | --- | --- | --- | --- |
| **Very easy** |  |  |  |  |  |  |  |  | **Very difficult** |

1. Do you have any other comments about the physical or practical aspects of taking the photographs? Please do not include identifiable data such as names

**General questions**

1. Having completed the study, do you think being asked to photograph your digital lesion/ulcer once (1x) per day is: *(please tick one)*

| Far too often (prefer once a week or less) | 🗆 |
| --- | --- |
| I’d rather do it less often (2-3 times per week) | 🗆 |
| Fine for me! | 🗆 |

1. Would you be willing to use a mobile phone app to record information about your digital lesions/ulcers in the future? *(please tick one)*

| Yes | 🗆 |
| --- | --- |
| No | 🗆 |

**END OF QUESTIONNAIRE**
