# Supplementary material for: Feasibility study of mobile phone photography as a possible outcome measure of systemic sclerosis-related digital lesions
Source: Rheumatol Adv Pract. 2022 Dec 7;6(3):rkac105. doi: 10.1093/rap/rkac105 (PMC9757677; doi:10.1093/rap/rkac105)
Supplement: rkac105_Supplementary_Data [file rkac105_supplementary_data.zip › S1_Smartphone_usage_questionnaire.doc]

**Development of a measuring app for finger lesions as an outcome measure for systemic sclerosis-related digital ulceration (SALVE: Scleroderma App for Lesion VErification) (Study 1)**

**Smartphone questionnaire**

**Please answer the following questions by placing either a tick or a cross in the boxes, like so: [] or []**

| **1. What is your age?** | 18 – 24 [ ]  25 – 34 [ ]  35 – 44 [ ]  45 – 54 [ ]  55 – 64 [ ]  65+ [ ] | |
| --- | --- | --- |
| **2. Do you own a mobile phone?** | Yes [ ] | No [ ] |
| **2a. If “Yes” above, what is the make/model of your phone?** |  | |
| **3. Can you take pictures (photographs) with your phone?** | Not sure [ ]  No, it doesn’t have a camera [ ]  It has a camera, but I don’t know how to use it [ ]  It has a camera, and I have taken photographs in the past [ ] | |
| **4. Do you use your mobile for (tick all that apply):** | Calls [ ]  Texting [ ]  Picture messaging (MMS) [ ]  Internet/email [ ]  Games [ ]  Other apps [ ] | |
| **5. Is your ability to use a mobile phone impaired by your digital ulcers?** | Not at all [ ]  A little [ ]  A lot [ ]  I can’t use a mobile phone due to my condition [ ] | |
| **6. Would you be interested in using your mobile phone to capture information about your digital ulcers?** | Yes [ ]  No [ ]  I’m not sure [ ] | |
| **7. Do you have any other comments to make about your mobile phone or mobile usage? (Please do not include any identifiable data such as your name when completing the comments section)** |  | |
